# High vasopressor doses are associated with decreased tissue oxygenation in critically ill patients: a secondary analysis of a prospective cohort

**DOI:** 10.1186/s13054-026-06110-w

**Published:** 2026-05-29

**Authors:** Patrick Rehn, Katharina Hölzl, Silvia Seidlitz, Ayca von Garrel, Tobias Hölle, Maik von der Forst, Alexander Studier-Fischer, Mascha Fiedler-Kalenka, Dania Fischer, Felix CF Schmitt, Christoph Lichtenstern, Markus Alexander Weigand, Lena Maier-Hein, Maximilian Dietrich, Stephan Katzenschlager

**Affiliations:** 1 Department of Anesthesiology, Heidelberg University Hospital, Im Neuenheimer Feld 420, Heidelberg, 69120 Germany; 2 Division of Intelligent Medical Systems (IMSY), German Cancer Research Center (DKFZ), Im Neuenheimer Feld 280, Heidelberg, 69120 Germany; 3 Department of General, Visceral, and Transplantation Surgery, Heidelberg University Hospital, Im Neuenheimer Feld 420, Heidelberg, 69120 Germany; 4 Department of Urology and Urosurgery, Medical Faculty of the University of Heidelberg, University Medical Center Mannheim, Theodor-Kutzer-Ufer 1-3, Mannheim, 68167 Germany

**Keywords:** Hyperspectral, Microcirculation, Catecholamines, Shock, Perfusion, Vasopressor

## Abstract

**Background:**

Despite stabilizing macrocirculatory blood pressure, vasopressors may deleteriously affect microcirculatory perfusion in critically ill patients. As microcirculatory dysfunction is associated with adverse outcomes and objective bedside monitoring remains limited, hyperspectral imaging (HSI) has emerged as a promising noninvasive method to assess tissue oxygenation. This study investigated the association between load of vasoactive medication and microcirculatory impairment using HSI in critically ill patients.

**Methods:**

In this secondary analysis of the prospective HySpec-ICU study, 502 surgical ICU patients were included. HSI measurements of the hand were performed on the day of admission to determine tissue oxygenation (StO₂) and other HSI variables. Multivariable linear regression and mediation analysis were employed to investigate the association between Norepinephrine Equivalent (NEE) and StO₂ and its impact on serum lactate levels.

**Results:**

Higher NEE was independently associated with significantly lower StO₂ (B = − 0.0931, β=−0.193, *p* = 0.001), while MAP showed no significant correlation with StO₂. Patients in the highest NEE quartile (> 0.28) exhibited the lowest StO₂ and the highest 30-day mortality (41.8%). StO₂ partially mediated the relationship between vasopressor load and arterial lactate. StO₂ generally improved after shock reversal defined as NEE ≤ 0.05, lactate < 2mmol/l, MAP ≥ 65mmHg for at least 24 h (+ 5.8%, *p* < 0.001).

**Conclusion:**

High vasopressor requirements are associated with impaired microcirculatory oxygenation of the hand regardless of systemic blood pressure. HSI provides an objective bedside tool to monitor these alterations, potentially identifying patients with persistent microcirculatory shock who require intensified therapy beyond macrohemodynamic targets.

**Supplementary Information:**

The online version contains supplementary material available at 10.1186/s13054-026-06110-w.

## Background

In critically ill patients with circulatory shock, vasopressor therapy is frequently required to maintain adequate mean arterial pressure and tissue perfusion. With potent vasoactive medications, macrohemodynamic goals can often be achieved. However, tissue perfusion may deteriorate despite achieving a targeted mean arterial pressure (MAP) with high doses of vasoactive medication. These microcirculatory alterations can consequently impair tissue/organ function and, hence, the outcomes of critically ill patients [1, 2].

Currently, in addition to laboratory values and the patient’s clinical presentation, macrocirculatory parameters are used to guide the diagnosis and subsequent patient management. [3]. In healthy individuals, normal macrocirculatory function is generally associated with adequate microcirculation perfusion, a relationship referred to as hemodynamic coherence. However, in critically ill patients, this coherence may be disrupted, such that restored macrocirculation does not necessarily imply sufficient microcirculation [4, 5]. Septic shock is a prototypical example of impaired hemodynamic coherence, with microcirculatory dysfunction representing a central pathophysiological mechanism [6]. Despite normalization of macrocirculatory variables, it is often not possible to achieve sufficient perfusion at the microcirculatory level [6]. Consequently, increasing attention has been directed to assessing microcirculation, especially as a potential target parameter in hemodynamic therapy [7].

A limited number of parameters are currently available to assess microcirculation including biochemical markers (e.g., serum lactate), clinical scores (e.g., mottling score), and bedside tests like capillary refill time (CRT). While CRT is widely used due to its ease of conduct, it remains observer dependent, influenced by external factors and only provides indirect measurement of microcirculation [8–10]. This underscores the need for objective, non-invasive technologies such as hyperspectral imaging (HSI).

HSI is an emerging bedside technology for evaluating microcirculation. By leveraging the specific light-absorbance properties of hemoglobin, HSI quantifies tissue perfusion and microcirculation [11]. In noninvasive bedside applications, the skin serves as the primary measurement site. HSI has shown significant promise across various clinical and experimental settings [12–19]. Notably, a strong correlation between HSI-derived microcirculatory measurements of internal organs and the skin has been shown [12].

This study investigated hyperspectral imaging in critically ill patients, with a focus on the association between vasopressor therapy and microcirculation impairment. The primary hypothesis was that higher vasopressor doses are associated with lower tissue oxygenation, as assessed by hyperspectral imaging.

## Methods

### Study design and ethics

Methodology and results are reported according to STROBE-guidelines for cohort studies [20]. This study is a secondary analysis of the prospective HySpec-ICU-study [21]. In this prospective observational study, we collected HSI data and corresponding RGB images from the skin of patients admitted to the interdisciplinary surgical ICU at the University Hospital Heidelberg, Germany. All adult patients admitted between October 24, 2022, and December 15, 2023, were included. A total of 508 patients was initially included in the database. The study was conducted in accordance with the ethical standards laid down in the 1964 Declaration of Helsinki and its later amendments. The protocol was approved by the Ethics Committee of the Medical Faculty of Heidelberg University, Germany (study reference number: S-288/2022, Chairperson Prof. Dr. med. Dr. h.c. Thomas Strowitzki) on April 24th 2022 and registered with the German Clinical Trials Register (study identifier: DRKS00029709; date of registration: 22th August 2022) prior to the commencement of recruitment. The ethics committee deferred individual patient consent because HSI was performed in all patients as part of routine care during the study period. Furthermore, HSI is non-invasive and poses no risk or discomfort to the patient.

The palm and annular finger were selected as measurement sites for their easy accessibility and low melanin content. Primarily, the hand with intra-arterial access was avoided. Structured clinical data were collected alongside the HSI data on the day of admission, including demographics, vital signs, blood gas analysis results, therapy details (use of organ replacement therapies, ventilation parameters, and doses of administered vasopressors and inotropes), and laboratory results. Vasopressor load was quantified at study inclusion using Norepinephrine Equivalents (NEE), according to an adapted formula by Kotani et al.: norepinephrine dose (µg/kg/min) + epinephrine dose (µg/kg/min) + 2.5 × vasopressin dose (U/min) [22]. Of note, no patients received dopamine, phenylephrine, angiotensin-II, terlipressin, methylene blue, metaraminol, hydroxycobalamine, or midodrine. Patients were grouped according to NEE quartiles (1) 0.01–0.05, (2) 0.05–0.11, (3) 0.11–0.28, and (4) > 0.28. Additionally, a fifth group (Group 0) comprising patients without vasoconstrictor drug administration (NEE = 0) was added. NEE values for group allocation were calculated on the day of study inclusion using the highest doses of vasoactive medication within 24 h. Mortality was assessed through a follow-up 30 days after study inclusion. Acute Kidney Injury (AKI) was defined according to the KDIGO criteria [23]. Furthermore, the association of tissue oxygenation (StO₂) with shock and shock reversal were evaluated. Shock reversal was defined as follows: minimal vasoactive support (NEE ≤ 0.05), lactate < 2mmol/l, and mean arterial pressure (MAP) ≥ 65mmHg for at least 24 h.

### Hemodynamic treatment protocol

According to local standards of care, noradrenaline was used as the first line vasopressor aiming for a MAP of 65mmHg. If a dose of 0.25 µg/kg/min of noradrenaline was not sufficient, vasopressin was added. Fluid therapy was individualized according to dynamic measures of preload (Pulse Pressure Variation, PPV; Passive Leg Raising Test, PLR) and according to echocardiographic findings (Stroke Volume Variation, SVV; Kissing Papillary Sign). Epinephrine or dobutamine were added if left ventricular was function was deemed insufficient in echocardiographic evaluation.

### Hyperspectral imaging

The camera system used was the medical device-graded TIVITA^®^ 2.0 Surgery Edition (Diaspective Vision GmbH, Am Salzhaff, Germany). It features a push-broom design with a spectral resolution of approximately 5 nm, covering 100 spectral channels in the range of 500 nm to 1000 nm. The resulting HSI cubes have dimensions of 640 × 480 × 100 (width × height × number of spectral channels). The imaged area is approximately 16 × 11.5 cm, with an imaging distance of about 50 cm, maintained by an integrated distance calibration system. Image acquisition takes approximately 7 s. The system includes both an HSI and an RGB sensor, providing simultaneous RGB images with dimensions of 640 × 480 × 3 (width × height × number of channels). Based on this dataset, the following HSI parameters are calculated using their respective spectral wavelengths:

- Tissue oxygenation saturation (StO_2_) wavelength range: 500–650 and 700–815 nm, indicated in percent (0–100%), penetration depth of ≈ 1 mm.
- Near infrared perfusion index (NIR) wavelength range: 655–735 and 825–925 nm, indicated in predefined arbitrary units (0–100), penetration depth of ≈ 4–6 mm.
- Tissue hemoglobin index (THI) wavelength range: 530–590 and 785–825 nm, indicated in predefined arbitrary units (0–100).
- Tissue water index (TWI) wavelength range: 880–900 and 955–980 nm indicated in predefined arbitrary units (0–100).

These tissue parameter images are estimated from the HSI data according to the formulas published by Holmer et al. [24].

To improve reproducibility in a clinical setting, all window blinds were lowered, and all light sources except the integrated light-emitting diode (LED) unit were turned off. No additional precautions were taken. The examiner supported patients’ hands to prevent motion artifacts and ensure more uniform hand positioning, using a consistent background across all images. Despite using a uniform background and standardizing hand positioning as much as possible, images might still include elements such as dressings, wounds, wires, tubes, or parts of the examiner’s gloved hand. To mitigate potential confounding from these elements, our analysis was performed on annotated skin areas. We chose circular annotations to consistently capture the same measurement sites across patients, regardless of hand rotation in the imaging plane. According to our annotation guidelines, the selected annotation radii were 100 pixels for the palm and 20 pixels for the ring finger. Finger annotations were centered on the fingertip, and palm annotations were centered on the palm of the hand, defined as the area enclosed by the wrist, the metacarpophalangeal joints, and the thumb basal joint. In this study, HSI data stemming from the pictures taken at the palm were used. Measurement variability can be up to 11% for StO_2_ according to our prior work [19].

### Sepsis definition

Diagnosis of sepsis was based on the Sepsis-3 criteria, which define it as acute, life-threatening organ dysfunction resulting from a suspected or confirmed infection [25]. Organ dysfunction was evaluated using the SOFA score, with an acute increase of at least two points indicating sepsis. Septic shock was defined as hypotension requiring vasopressors to maintain a mean arterial pressure of ≥ 65mmHg and a lactate level of ≥ 2 mmol/l (18 mg/dl) despite adequate volume resuscitation [25]. Differentiating between organ failure caused by sepsis and that resulting from non-septic inflammation can be challenging, particularly in a surgical ICU setting following surgical trauma. To maintain label quality and avoid ambiguity in such cases, we introduced a third label, “unsure”, alongside the labels “sepsis” and “no sepsis”. For each patient, the sepsis status was independently assessed by two expert anesthetists. Disagreements between the two anesthetists were resolved by a third, more senior anesthetist (the head of the department for anesthesia and intensive care).

### Endpoints

The primary endpoint was the relationship of NEE on StO₂, assessed using hyperspectral imaging. Secondary endpoints included the relationship of NEE on near infrared spectroscopy index (NIR), tissue water index (TWI), tissue hemoglobin index (THI), lactate levels, and renal function assessed by creatinine levels. Additionally, differences in StO₂ before and after shock reversal were evaluated. Also, 30-day survival (post inclusion) was assessed. For secondary endpoints, patients were divided into 2 groups according to their StO₂ value on day 1. Cut point for these groups was the median StO₂ of 0.58 (“Good StO₂”: StO₂ ≥ 0.58; “Poor StO₂”: StO₂ < 0.58).

### Usage of artificial intelligence tools

The authors acknowledge the use of Generative AI tools and associated ethical responsibilities. ChatGPT (OpenAI, San Francisco, USA) was used to improve grammar and language coherence. All content was subsequently reviewed and edited by the authors to ensure accuracy, integrity, and compliance with publication standards. The authors assume full responsibility for the final manuscript.

### Statistical methods

Continuous data are presented as mean and standard deviation (SD). Categorical parameters are reported as absolute and relative frequencies. Comparisons of continuous variables across groups were performed using one-way analysis of variance (ANOVA). Post hoc pairwise comparisons were performed using Tukey’s test when the ANOVA indicated a significant difference. Categorical outcomes (mortality, shock reversal) were compared using the Fisher’s exact test. The Cochran-Armitage trend test was used to assess whether 30-day survival followed a linear downward trend across NEE groups. Odds ratios (OR) and risk ratios (RR) with 95% confidence intervals were calculated for all binary outcomes using the Wald method. To examine the association between NEE levels and tissue oxygenation, we conducted a multivariable linear regression analysis with StO₂ as the dependent variable. The independent variables included arterial lactate concentration (mmol/L), age (years), MAP (mmHg), the Acute Physiology And Chronic Health Evaluation (APACHE) II-Score, and NEE. All predictors were entered simultaneously using the standard enter method. Model assumptions were assessed. Variance inflation factors (VIFs) were used to evaluate multicollinearity, with values > 5 considered indicative of problematic collinearity (all VIFs < 2). The residuals were approximately normally distributed, as evidenced visually by the histogram and Q-Q plot, despite significant formal tests likely influenced by the large sample size. Residual plots indicated potential heteroscedasticity, particularly for lower predicted values, although no influential cases were detected (max Cook’s D = 0.08). Given these findings, we retained the linear model for interpretability, while acknowledging modest deviations from model assumptions. To address residual heteroscedasticity identified in diagnostic plots, we performed a sensitivity analysis using heteroscedasticity-consistent (HC) standard errors with the HC3 correction. All key findings remained statistically significant after HC3 correction.

To assess whether the association between NE dose and StO₂ departed from linearity, we extended the multivariable linear regression model by additionally fitting a quadratic term and a restricted cubic spline for NEE, while adjusting for lactate (mmol/L), age (years), MAP (mmHg), and SOFA score. Non-linearity was assessed by the significance of the quadratic term, the non-linear spline component, and the comparison of model fit with the original linear model.

To evaluate whether alterations in tissue oxygenation mediated the relationship between vasopressor requirements and markers of tissue hypoperfusion, a mediation analysis was performed using the PROCESS macro for SPSS, version 4.2 (Andrew F. Hayes, www.processmacro.org) [26, 27]. In this model, the NEE was entered as the independent variable (X), skin tissue oxygenation (StO₂) as the mediator (M), and serum lactate concentration as the dependent variable (Y), while mean arterial pressure, SOFA and age were included as covariates. The analysis used PROCESS Model 4 (simple mediation) with 5000 bootstrap samples and a 95% bis-corrected confidence interval (CI) for the indirect effect. Significance of mediation was inferred when the bootstrap CI for the indirect path did not include zero.

A p-value < 0.05 was considered statistically significant. All analyses were performed using R version 4.5.1. (R Foundation for Statistical Computing, Vienna, Austria), SPSS version 29.0 (IBM^®^), and Python 3.9 with scipy and scikit-learn libraries.

## Results

### Baseline characteristics

A total of 508 patients were screened (Hyspec ICU cohort). We excluded 6 patients due to missing data on vasopressor use on day 1 (study inclusion), leaving 502 patients for the final analysis. Baseline characteristics of excluded patients are reported in Supplemental Table 1. Patients were categorized into five groups based on NEE quartiles on day 1, with an additional group comprising patients who did not receive vasopressors on day 1 (NEE = 0) (Table 1).

**Table 1:** baseline characteristics for whole cohort (*n* = 502)

Variable	NEE 0 (*n* = 221)	NEE 0.01–0.05 (*n* = 71)	NEE 0.05–0.11 (*n* = 70)	NEE 0.11–0.28 (*n* = 70)	NEE > 0.28 (*n* = 70)
Age, years, mean ± SD	62.1 ± 14.3	62.8 ± 13.3	65.3 ± 14.2	66.0 ± 14.1	63.3 ± 16.5
Weight, mean ± SD [kg]	80.9 ± 18.8	85.9 ± 21.2	83.5 ± 23.2	80.8 ± 27.2	77.5 ± 23.6
Female sex, n (%)	63 (28)	15 (21)	23 (33)	28 (40)	18 (26)
APACHE II score, mean ± SD	16.7 ± 4.7	18.4 ± 6.5	20.5 ± 6.2	26.3 ± 6.9	30.7 ± 6.4
SOFA score, mean ± SD	4.2 ± 2.6	7.2 ± 3.1	8.3 ± 3.2	11.3 ± 2.7	12.9 ± 2.6
Preexisting cardiovascular disease, n (%)	103 (48)	29 (41)	35 (52)	30 (46)	29 (47)
CKD, n (%)	31 (14)	10 (15)	13 (19)	11 (16)	18 (26)
Acute kidney injury, n (%)	50 (23)	14 (20)	19 (27)	50 (71)	42 (60)
RRT, n (%)	12 (5)	2 (3)	6 (9)	9 (13)	20 (29)
Malignant disease, n (%)	92 (42)	35 (49)	29 (41)	27 (40)	20 (29)
Ventilated at baseline, n (%)	36 (16)	18 (25)	31 (44)	50 (71)	64 (91)
Fluid Balance Day 1, mean ± SD [ml]	1158.2 ± 2462.9	1407.9 ± 1836.5	2807.3 ± 8574.3	2512.4 ± 3063.4	2822.9 ± 3567.3
Sepsis present, n (%)	14 (6)	9 (13)	21 (30)	32 (46)	50 (71)
Septic shock, n (%)	3 (1)	1 (1)	1 (1)	19 (27)	26 (37)
Unclear Sepsis Status, n (%)	24 (11)	5 (7)	4 (6)	13 (9)	7 (10)
Sepsis focus (among sepsis patients)					
Abdominal, n (%)	7 (50)	4 (44)	11 (52)	18 (56)	29 (58)
Pulmonary, n (%)	1 (7)	2 (22)	4 (19)	7 (22)	9 (18)
UTI, n (%)	1 (7)	1 (11)	1 (5)	0 (0)	0 (0)
Soft tissue/skin, n (%)	1 (7)	2 (22)	0 (0)	2 (6)	2 (4)
Multiple foci, n (%)	2 (14)	0 (0)	4 (19)	2 (6)	3 (6)
Other/unknown, n (%)	2 (14)	0 (0)	1 (5)	3 (9)	7 (14)

Values given as mean and standard deviation or absolute and relative frequencies. APACHE: Acute Physiology And Chronic Health Evaluation-Score, SOFA: Sequential Organ Failure Assessment Score, CKD: Chronic Kidney Failure, RRT: Renal Replacement Therapy, UTI: Urinary Tract Infection. NEE: norepinephrine-equivalent dose (µg/kg/min)

### Primary outcome

**Fig. 1:**
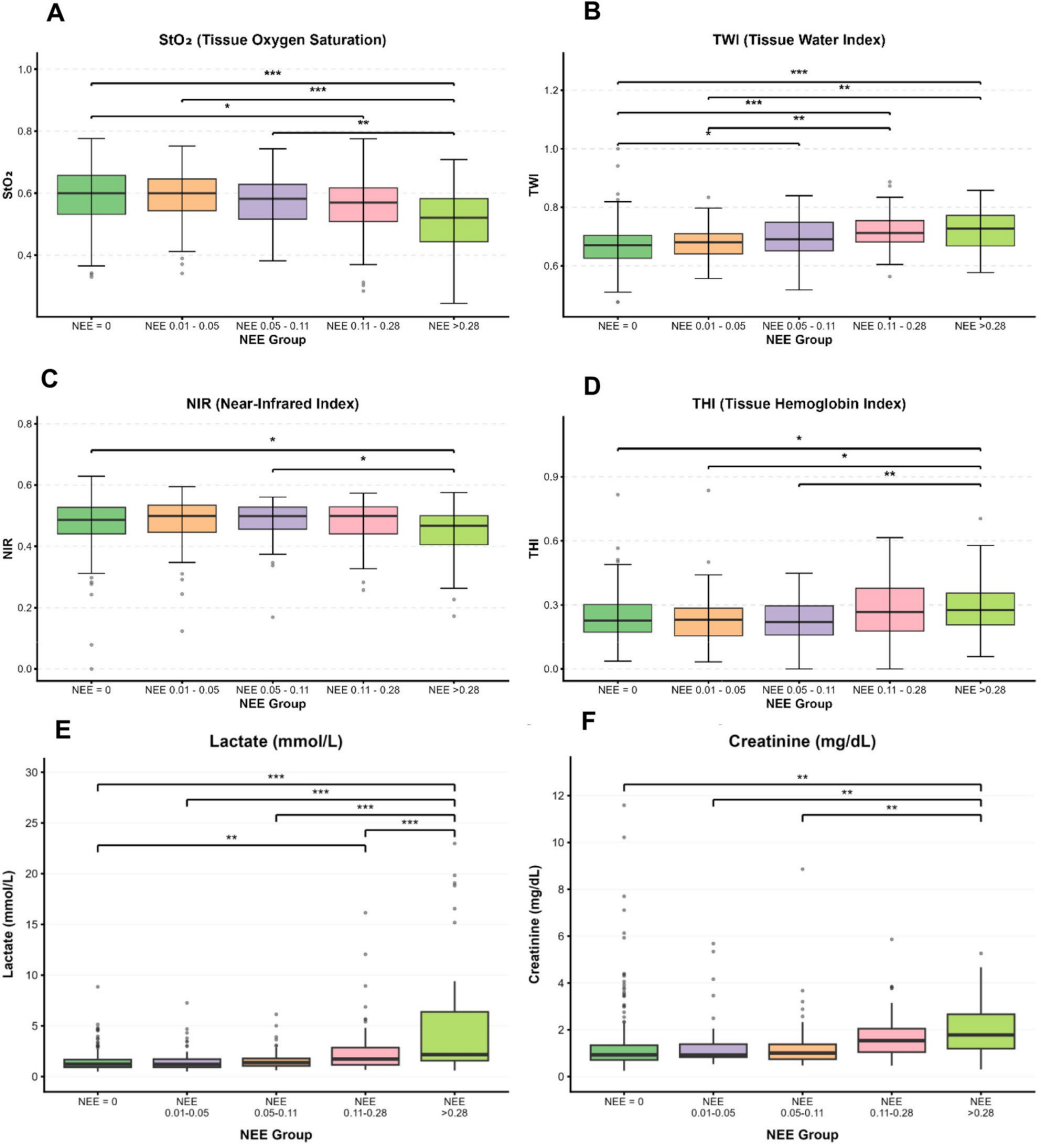
Boxplots for hyperspectral imaging data and laboratory values across NEE groups on day of inclusion of whole cohort. **A**: StO2, **B**: TWI, **C**: NIR, **D**: THI, **E**: Lactate, **F**: Creatinine. The box represents the interquartile range (IQR, 25th–75th percentile), the horizontal line indicates the median, and the whiskers extend to 1.5×IQR; values beyond this range are shown as outliers. StO₂ = tissue oxygenation index in [%], NIR = near infrared spectroscopy index (arbitrary index), TWI = tissue water index (arbitrary index), THI = tissue hemoglobin index (arbitrary index), NEE: Norepinephrine Equivalent (µg/kg/min). * = *p* < 0.05, ** = *p* ≤ 0.01, *** = *p* < 0.001

The primary outcome was analyzed at the time of study inclusion (day 1), with significant differences across NEE groups for StO₂ (*p* < 0.001), NIR (*p* = 0.030), TWI (*p* < 0.001), THI (*p* = 0.002), lactate levels (*p* < 0.001), and creatinine levels (*p* < 0.001). Post-hoc pairwise Tukey’s tests showed that patients in Group 4 (NEE > 0.28) had significantly lower StO₂ than those in Group 0 (NEE 0) and Group 1 (NEE 0.01–0.05) (Fig. 1, StO_2_). Further, significantly higher TWI was observed in Group 0 compared with Groups 3 and 4 (Fig. 1, TWI), with lactate and creatinine values also higher than those in the lower NEE groups. Full results are shown in Supplemental Table 2 and Fig. 1.

In the adjusted linear regression model, a higher NEE dose was independently associated with lower StO₂ (B = −0.093, 95% CI −0.150 to −0.036, *p* = 0.001), whereas lactate and age were also negatively associated with StO₂, and MAP and SOFA were not (Table 2). To assess whether this relationship departed from linearity, we additionally fitted a quadratic term and a restricted cubic spline for NEE. The quadratic term was not significant (*p* = 0.450), and the restricted cubic spline analysis showed no evidence of a non-linear component (*p* = 0.693) (Supplemental Table 3). Model fit was not improved by the quadratic or spline specifications compared with the linear model. These findings support the use of a linear term to describe the association between NEE and StO₂ in the present cohort.

**Table 2:** Linear regression model predicting tissue oxygenation (StO₂)

Predictor	B (SE)	95% CI	β	t	*p*
Age (years)	−0.0008 (0.0003)	[−0.0013, −0.0002]	−0.114	−2.69	0.007
MAP (mmHg)	0.0002 (0.0003)	[−0.0004, 0.0008]	0.029	0.50	0.503
Lactate (mmol/L)	−0.00086 (0.0003)	[−0.0013, −0.0004]	−0.204	−4.08	< 0.001
SOFA	−0.0007 (0.0012)	[−0.0031, 0.0016]	−0.033	−0.60	0.546
NEE	−0.0931 (0.0288)	[−0.1496, 0.0366]	−0.193	−3.23	0.001

Model statistics: R² = 0.154, Adjusted R² = 0.146, F(6,474) = 17.32, *p* < 0.001. B = unstandardized regression coefficient; SE = standard error; CI: Confidence Interval; β = standardized regression coefficient; t = t statistic; MAP: Mean Arterial Pressure, NEE: Norepinephrine Equivalent (µg/kg/min). SOFA: Sequential Organ Failure Assessment Score

**Fig. 2:**
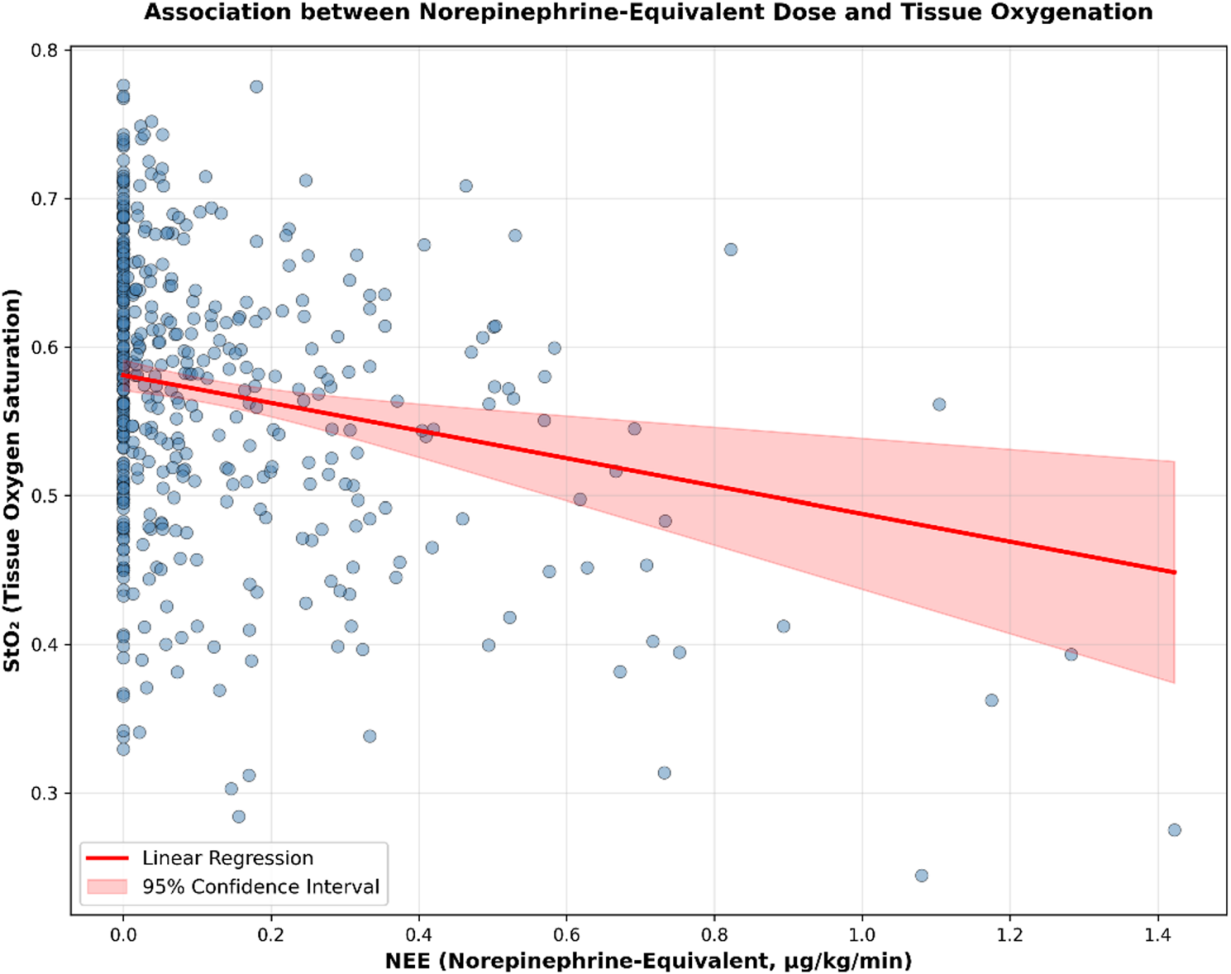
Association between Norepinephrine Equivalent (NEE) and tissue oxygen saturation (StO₂). Scatter plot illustrates the relationship between the systemic vasopressor load (NEE) and peripheral microcirculation (StO₂) measured via hyperspectral imaging. The red line represents the linear regression, showing a weak negative correlation between increasing NEE and StO₂ level (*r* = −0.327, *p* < 0.001). The shaded area indicated the 95%-Confidence Interval

Inspection of residual plots indicated minor heteroscedasticity, predominantly at lower predicted values of tissue oxygenation. Residuals were approximately normally distributed, and Cook’s distance showed no influential observations (max Cook’s D < 0.1, Supplemental Fig. 1–3). Sensitivity analysis using HC3 robust standard errors yielded substantively identical results. The association between NEE and StO₂ remained highly significant (*p* = 0.004). Thus, the assumptions of linear regression were considered adequately met (Supplemental Figs. 1–3, Supplemental Table 4). Both arterial lactate concentration (B = − 0.0086, β = − 0.204, *p* < 0.001) and NEE (B = − 0.0931, β = − 0.193, *p* = 0.004) were independently associated with lower tissue oxygenation. StO₂ decreased by 0.009 units per 1 mmol/L increase in lactate and by 0.093 units per 1-unit increase in NEE. Age showed a smaller but significant negative association (B = − 0.0008, β = − 0.1114, *p* = 0.007), while MAP was not significantly related to StO₂ (Table 3). NEE and StO₂ showed a weak negative correlation (*r* =−0.327, *p* < 0.001; Fig. 2).

**Table 3:** Mediation model depicting the association between vasoactive–inotropic score (NEE) (X), tissue oxygenation (StO₂) (M), and lactate (Y)

Path	Outcome variable	Predictors	B (unstandardized)	SE	95% CI	t	*p*	Standardized β
a	StO₂	NEE	−0.139	0.0270	−0.192 to −0.086	–5.14	< 0.001	−0.287
		MAP	0.0003	0.0003	−0.0003 to 0.0009	0.88	0.377	0.049
		Age	−0.0008	0.0003	−0.001 to −0.0002	–2.80	0.005	−0.120
		SOFA	−0.0011	0.0012	−0.004 to 0.0013	−0.93	0.355	−0.051
b, c′	Lactate	NEE	47.714	5.860	36.229 to 59.199	8.14	< 0.001	0.415
		StO₂	−39.568	9.691	−58.563 to −20.573	−4.08	< 0.001	−0.166
		MAP	−0.070	0.065	−0.198 to 0.059	−1.06	0.288	−0.042
		Age	0.017	0.060	−0.101 to 0.135	0.28	0.779	0.011
		SOFA	0.425	0.259	−0.083 to 0.933	1.64	0.101	0.081
c (total)	Lactate	NEE	53.206	5.797	41.844 to 64.567	9.18	< 0.001	0.462
		MAP	−0.081	0.067	−0.211 to 0.050	−1.21	0.227	−0.048
		Age	0.048	0.061	−0.071 to 0.168	0.79	0.427	0.031
		SOFA	0.470	0.263	−0.045 to 0.986	1.79	0.075	0.089
Indirect effect (a × b)	Lactate	NEE (via StO₂)	5.492	1.718	2.181 to 9.038	—	—	—

Path a represents the effect of NEE on StO₂; path b the effect of StO₂ on lactate controlling for NEE; path c the total effect of NEE on lactate; and path c′ the direct effect of NEE on lactate after accounting for StO₂. The indirect effect (a × b) quantifies the mediated component. Sample size *n* = 502. CI: Confidence Interval, MAP: Mean Arterial Pressure, StO₂: tissue oxygen saturation, NEE: Norepinephrine Equivalent (µg/kg/min)

The exploratory mediation analysis showed higher NEE was significantly associated with lower StO₂ (B = − 0.139, 95% CI −0.192 to −0.086, *p* < 0.001). Lower StO₂ was in turn significantly associated with higher lactate levels after adjustment for NEE (B = −39.57, 95% CI −58.563 to −20.573, *p* < 0.001). The total effect of NEE on lactate was significant (B = 53.206, 95% CI 41.844 to 64.567, *p* < 0.001), and the direct effect of NEE on lactate remained significant after inclusion of StO₂ in the model (B = 47.714, 95% CI 36.229 to 59.199, *p* < 0.001). The indirect effect of NEE on lactate was partially mediated by StO₂ (B = 5.492, 95% CI 2.181 to 9.038), accounting for approximately 10.3% of the total effect. All variables were measured simultaneously; this represents cross-sectional associations. Full results of the mediation analysis are presented in Table 3.

In the subgroup of septic patients (*n* = 126), hyperspectral imaging parameters were compared across five groups defined by NEE quartiles, calculated specifically for this cohort, with an additional group including patients who did not receive vasopressor therapy (NEE = 0). A significant difference was observed in median StO₂ among groups (*p* = 0.013), with post-hoc analysis showing lower StO₂ in patients with NEE > 0.38 compared to those without vasopressor therapy (mean difference = 0.107, *p* = 0.013). No significant group differences were found for NIR (*p* = 0.381), TWI (*p* = 0.507), or THI (*p* = 0.338). Full results are shown in Table 4. The overall effect size for StO₂ differences across NEE groups was small to moderate (η² = 0.098), whereas other hyperspectral parameters showed small or negligible effects (η² < 0.04). In the subgroup of non-septic patients significant difference between patients without vasopressors (NEE = 0) and patients in the with the highest vasopressor requirements (NEE > 0.16) persisted (0.588 vs. 0.537, *p* = 0.011). Results for the non-sepsis subgroup are presented in the Supplements (Supplemental Table 5).

**Table 4:** Sepsis subgroup, post-hoc tukey test: significant difference for StO₂ between NEE = 0 and NEE > 0.38 (*p* = 0.013)

Parameter	NEE = 0 (*n* = 14)	NEE 0.01 − 0.11 (*n* = 28)	NEE 0.11 − 0.25 (*n* = 28)	NEE 0.25–0.38 (*n* = 28)	NEE > 0.38 (*n* = 28)	*p*-value (ANOVA)
StO₂, mean ± SD	0.609 ± 0.11	0.569 ± 0.10	0.552 ± 0.10	0.529 ± 0.08	0.501 ± 0.12	0.013
NIR, mean ± SD	0.499 ± 0.07	0.456 ± 0.11	0.475 ± 0.09	0.456 ± 0.08	0.446 ± 0.09	0.381
TWI, mean ± SD	0.690 ± 0.07	0.724 ± 0.06	0.720 ± 0.06	0.723 ± 0.07	0.724 ± 0.07	0.507
THI, mean ± SD	0.247 ± 0.12	0.285 ± 0.14	0.298 ± 0.15	0.271 ± 0.09	0.327 ± 0.13	0.338

StO₂: tissue oxygen saturation, NIR: near-infrared index, TWI: tissue water index, THI: tissue hemoglobin index. NEE: Norepinephrine Equivalent (µg/kg/min)

### Secondary outcomes

30-day mortality was available for 478 patients and increased across increasing NEE categories, from 4.8% in the vasopressor-free group (NEE = 0) to 41.8% in the highest NEE group (Chi-squared *p* < 0.001). A significant inverse linear trend was observed across NEE groups for 30-day mortality (Cochran-Armitage trend test: Z = 7.56, *p* < 0.001) (Suppl. Figure 4). This analysis aims to describe outcomes across strata of circulatory support intensity within the study cohort. In the sepsis cohort (*n* = 116 available for survival analysis) mortality differed from 14.3% in the vasopressor free group (NEE = 0) to 38.5% in the highest NEE group. Statistical significance was not reached (Chi-squared *p* = 0.430).

In patients with shock (NEE > 0 and lactate ≥ 2 mmol/l), 30-day survival was not significantly different between those with below-median StO₂ at day 1 and those with above-median StO₂ at day 1 (56.1% vs. 67.7%; *p* = 0.375).

**Fig. 3:**
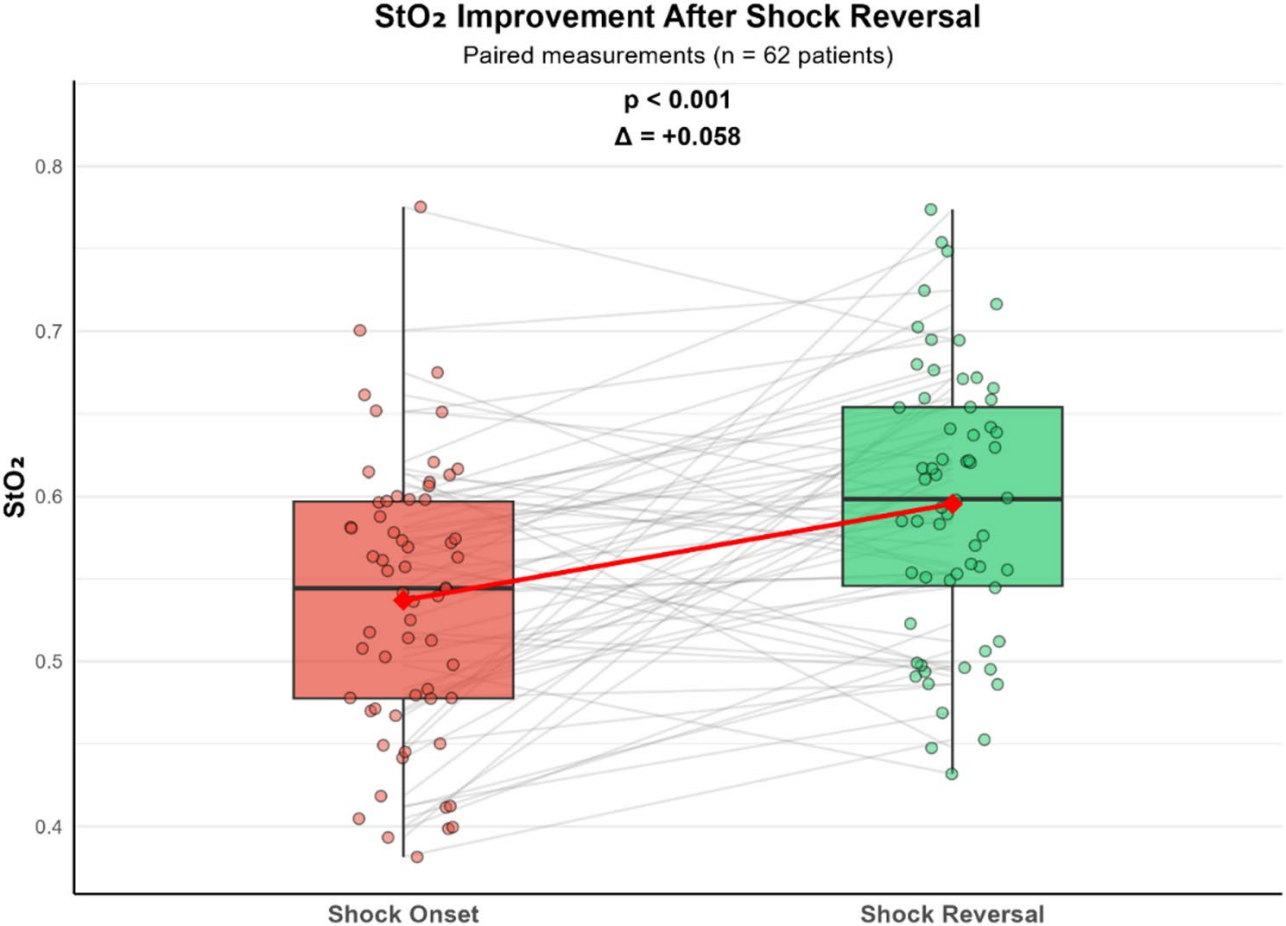
Change in StO₂ in from shock onset to reversal. Red diamonds represent mean values (shock 0.537, reversal 0.595). Despite overall improvement (+ 5.8%, *p* < 0.001), substantial heterogeneity was observed: 45 patients (72.6%) showed improvement while 17 patients (27.4%) demonstrated worsening, with changes ranging from − 16.7% to + 32.5%. Each thin gray line represents one individual patient. Box plots show median and interquartile range; individual data points are displayed as dots

A total of 62 patients with shock at any time point and recorded shock reversal were further analyzed. StO₂ improved significantly after shock reversal (mean difference + 0.058, 95% CI 0.033–0.084, *p* < 0.001; Fig. 3). Patients were then stratified according to improvement, worsening, or no change in StO₂ from shock onset to shock reversal. Patients who had improved StO₂ values at shock reversal had worse StO₂ at shock onset (0.512 ± 0.076 vs. 0.602 ± 0.067, *p* < 0.001). For 61 patients, complete mortality data were available. In this cohort, 30-day mortality was 13% (6/45) in patients with improved StO₂, compared with 25% (4/16) in those with worsened/unchanged StO₂ (Fisher’s exact test, *p* = 0.431). The OR was 2.17 (95% CI 0.52–8.97)and RR was 1.88 (95% CI 0.61–5.80). The increase in values did not differ significantly between survivors and non-survivors, despite both groups achieving shock reversal (mean difference + 0.066 vs. +0.031, *p* = 0.358; Supplemental Fig. 5).

## Discussion

In this secondary analysis of a large prospective cohort of critically ill ICU patients, increasing vasoactive support, as indicated by higher NEE, was associated with impaired microcirculatory tissue oxygenation, as measured by HSI. This association remained significant after adjustment for age and MAP. Moreover, our mediation analysis showed that impaired tissue oxygenation partially mediated the relationship between NEE and serum lactate levels, suggesting microcirculatory compromise as one mechanistic link between high vasopressor exposure and metabolic derangement. These findings suggest that higher vasopressor requirements, as reflected by NEE, are not merely surrogates of systemic hemodynamics but represent an independent factor associated with reduced microcirculatory oxygen delivery. This study provides adds to our understanding of the microcirculatory effects of vasoactive therapy, addressing a significant research gap recently identified by Sathianathan et al [28].

In recent years, the concept of hemodynamic coherence has gained increasing attention. Under physiological conditions, macrocirculatory parameters reliably reflect the status of microcirculation. In critically ill patients, however, this relationship may be disrupted. These patients are “hemodynamically incoherent” as macrohemodynamic parameters do not necessarily reflect microcirculatory tissue perfusion and oxygenation [4–6, 29, 30]. Several studies have shown that normalizing macrohemodynamics using vasoactive agents can be misleading [31–33]. Our data extend this body of work by quantifying a dose–response relationship between vasopressor load and cutaneous microvascular oxygenation in a critically ill population.

The findings from the ANDROMEDA-SHOCK-2 trial reinforce the need for a microcirculation- and perfusion-guided approach [8, 9]. In the context of our findings, where higher vasopressor load was associated with worse cutaneous tissue oxygenation and higher lactate, the ANDROMEDA-SHOCK-2 data underscore the notion that a perfusion-guided strategy may attenuate the detrimental microcirculatory consequences of aggressive vasopressor escalation. In the septic subgroup of our study, the NEE–StO₂ relationship remained robust, suggesting that vasoplegia and endothelial dysfunction characteristic of sepsis amplify the microvascular vulnerability to high vasopressor doses. This is consistent with the narrative that sepsis is fundamentally a microcirculatory disease and that microvascular abnormalities correlate with organ dysfunction severity and mortality [1, 34, 35].

In states of distributive shock, where autoregulatory microvascular mechanisms are already compromised, high vasopressor doses may further impair tissue oxygen delivery and extraction [33, 36]. Our exploratory mediation results provide additional nuance. Because NEE, StO₂, and lactate were measured simultaneously, the mediation describes cross-sectional associations. Nevertheless, the finding that StO₂ mediated about 10% of the effect is consistent with the hypothesis that microvascular impairment contributes to the pathway leading to metabolic stress and lactatemia. Yet, the fact that a significant direct effect of NEE on lactate remained indicates that elevated lactate is multifactorial. Impaired hepatic clearance, β-adrenergic stimulated glycolysis, mitochondrial dysfunction and microvascular hypoxia also play an important role [37–42]. Therefore, while impaired microcirculation appears to contribute, it is not the sole explanation for elevated lactate in shock. These exploratory findings should therefore be interpreted as hypothesis-generating.

In an exploratory analysis of patients meeting criteria for shock reversal, mean StO₂ increased significantly, although trajectories varied substantially between individuals. ååPatients with stable or worsening StO₂ had an approximately two-fold higher 30-day mortality risk than those with improving StO₂, while survivors showed numerically greater StO₂ improvement than non-survivors; however, these associations were imprecise and limited by statistical power. Given the small sample size and exploratory nature of this analysis, these findings should be considered hypothesis-generating and validated in larger prospective studies.

Beyond tissue oxygenation, further HSI–derived parameters (TWI, THI, and NIR) showed meaningful patterns across NEE groups and support the interpretation that escalating vasopressor requirements reflect deeper microcirculatory disturbance. The increase in TWI with rising NEE suggests progressive tissue edema, consistent with the endothelial dysfunction, glycocalyx degradation, and capillary leak described in advanced shock states [43–45]. Prior microvascular studies have demonstrated that vasopressor-dependent shock is frequently accompanied by interstitial fluid accumulation and impaired lymphatic drainage, both of which further hinder oxygen diffusion at the tissue level [19, 46–48]. Similarly, the increase in THI in higher NEE groups likely reflects rising microvascular congestion and heterogeneity of red blood cell distribution. Several studies using direct videomicroscopy in hemodynamic shock observed this phenomenon, which is associated with impaired capillary recruitment and reduced functional capillary density [1, 2, 49–52]. In contrast, the decline in NIR in the highest NEE category aligns with reduced deeper-dermal perfusion, reinforcing that microvascular flow abnormalities extend beyond the superficial tissue layer captured by StO₂ alone. Collectively, these patterns provide converging physiological evidence of capillary flow derangements, venous stagnation, edema-related diffusion limitation, and redistribution of intratissue hemoglobin.

The clinical relevance of the observed 7.8%-point StO₂ difference between the NEE groups 0 and 4 merits consideration. While no universally established minimum clinically important difference (MCID) exists for HSI-derived StO₂, several lines of evidence support the meaningfulness of this magnitude. First, there are reported differences in StO₂ of about 10% between clinically relevant groups [19, 53, 54]. Second, the observed values range from low-normal (58.9%, NEE Group 0) to (51.1%, NEE Group 4), compared with the non-critically ill subjects’ range of 60–85% reported in several studies [11, 13, 19]. Third, the prognostic association and mechanistic link to lactate support that this magnitude may reflect clinically meaningful alterations in tissue oxygenation. Finally, the similarity to StO₂ changes observed with shock reversal suggests that differences of this scale are associated with hemodynamic recovery. Device-specific MCID validation is needed; given the preliminary evidence, differences in StO_2_ of 8% may distinguish clinically distinct perfusion states in vasopressor-dependent critically ill patients.

Our study has several strengths. First, it includes a relatively large ICU cohort with standardized HSI measurement of cutaneous oxygenation, adding objective microcirculatory data to the catecholamine–lactate axis. To reduce the impact of ambient light while ensuring applicability to other ICUs, window blinds were closed, and external lighting was turned off. Second, the use of mediation modeling enables insight into mechanistic pathways rather than simple associations. Third, adjusting for macrohemodynamic variables strengthens the argument that vasopressor dose has microvascular consequences beyond systemic perfusion.

However, there are important limitations. The observational nature of the study precludes causal conclusions. Moreover, the mediation analysis is limited by cross-sectional measurement of all variables at a single timepoint, preventing establishment of temporal precedence required for causal inference. High vasopressor dose may be a marker of more severe underlying illness rather than a direct mediator of microvascular injury. The use of NEE as a surrogate for exposure to vasoactive drugs does not discriminate between different agents or reflect duration precisely. Furthermore, it remains uncertain how well cutaneous microvascular assessment reflects perfusion in critical organs. Another important limitation is that our exploratory subgroup analyses (sepsis, shock reversal) were not prospectively powered and should be interpreted as hypothesis-generating. The wide confidence intervals in these smaller cohorts reflect statistical uncertainty and mandate cautious interpretation. Additionally, HSI has several inherent limitations. It is susceptible to interference from ambient light and is temperature-dependent [55]. To minimize these effects, image acquisition was standardized by lowering window blinds and turning off all external light sources, leaving only the integrated LED illumination active. Also, the room temperature was constant, and the windows were closed. The study was performed in a single surgical ICU, which may limit generalizability to non-surgical or medical populations. Finally, while we adjusted for key covariates, residual confounding cannot be excluded.

## Conclusion

In summary, our findings support the interpretation that increasing vasopressor requirements in critically ill patients are associated with impaired tissue oxygenation and rising lactate levels, mediated at least in part by microcirculatory dysfunction. These data reinforce the concept that vasopressor escalation, although often necessary for macrocirculatory stabilization, may compromise microcirculatory integrity and thereby limit effective tissue oxygen delivery. Non-invasive HSI of cutaneous oxygenation provides a promising adjunct to conventional monitoring by revealing microvascular “blind spots” that remain undetected by macrocirculatory parameters. Future prospective randomized trials should assess whether incorporating HSI-derived microcirculatory endpoints to guide vasopressor therapy can improve clinical outcomes.

## Supplementary Information

Supplementary Material 1.

## Data Availability

The datasets used and analyzed during the current study are available from the corresponding author on reasonable request.

## Abbreviations

APACHE: Acute physiology and chronic health evaluation-score
CI: Confidence interval
CKD: Chronic kidney disease
HSI: Hyperspectral imaging
ICU: Intensive care unit
LED: Light-emitting diode
MAP: Mean arterial pressure
NEE: Norepinephrine Equivalents
NIR: Near infrared spectroscopy index
OR: Odds Ratio
RGB: Red, green, blue
RR: Risk ratio
RRT: Renal replacement therapy
SD: Standard Deviation
SE: Standard Error
SOFA: Sequential organ failure assessment score
StO2: Tissue oxygenation index
THI: Tissue hemoglobin index
TWI: Tissue water index
UTI: Urinary tract infection
VIF: Variance inflating factor
