# Supplementary material for: High vasopressor doses are associated with decreased tissue oxygenation in critically ill patients: a secondary analysis of a prospective cohort
Source: Crit Care. 2026 May 29;30:277. doi: 10.1186/s13054-026-06110-w (PMC13221758; doi:10.1186/s13054-026-06110-w)
Supplement: Supplementary file 1 — Supplementary Material 1. [file 13054_2026_6110_MOESM1_ESM.docx]

**Supplemental Material to**

**High vasopressor doses are associated with decreased tissue oxygenation in critically ill patients**

Patrick Rehn^1^, Katharina Hölzl^1^, Silvia Seidlitz^2^, Ayca von Garrel^1^, Tobias Hölle^1^, Maik von der Forst^1^, Alexander Studier-Fischer^2,3^, Mascha Fiedler-Kalenka^1^, Dania Fischer^1^, Felix CF Schmitt^1^, Christoph Lichtenstern^1^, Markus Alexander Weigand^1^, Lena Maier-Hein^2^, Maximilian Dietrich^1^, Stephan Katzenschlager^1,#^

^1^ Department of Anesthesiology, Heidelberg University Hospital, Im Neuenheimer Feld 420, 69120 Heidelberg, Germany.

^2^ Division of Intelligent Medical Systems (IMSY), German Cancer Research Center (DKFZ), Im Neuenheimer Feld 280, 69120 Heidelberg, Germany

^3^ Department of General, Visceral, and Transplantation Surgery, Heidelberg University Hospital, Im Neuenheimer Feld 420, 69120 Heidelberg, Germany

^4^ Department of Urology and Urosurgery, Medical Faculty of the University of Heidelberg, University Medical Center Mannheim, Theodor-Kutzer-Ufer 1-3, 68167 Mannheim, Germany.

**^#^Corresponding author:**

Dr. med. Dr. med. univ. Stephan Katzenschlager

Stephan.Katzenschlager@med.uni-heidelberg.de

Department of Anesthesiology, Heidelberg University Hospital

Im Neuenheimer Feld 420

69120 Heidelberg, Germany

Table of Contents

[Supplemental Figure 1: Distribution of residuals. 3](#_Toc230285526)

[Supplemental Figure 2: Scatterplot of Residuals vs. Predicted Values 4](#_Toc230285527)

[Supplemental Figure 3: Scatter Plot of Cook's Distance 5](#_Toc230285528)

[Supplement Figure 4: 30-Day Mortality across NEE groups 6](#_Toc230285529)

[Supplement Figure 5: StO₂ trajectories from shock onset to reversal stratified by 30-day mortality 7](#_Toc230285530)

[Supplemental Table 1: Baseline Characteristics of excluded patients 8](#_Toc230285531)

[Supplemental Table 2: Hyperspectral Imaging data, lactate, and creatinine levels according to NEE-quartiles. 9](#_Toc230285532)

[Supplemental Table 3: Sensitivity analyses assessing non-linearity of the association between NEE and StO₂ 10](#_Toc230285533)

[Supplemental Table 4: Comparison of Standard Errors HC3 and OLS for Linear Regression 11](#_Toc230285534)

[Supplemental Table 5: Non-Sepsis Subgroup 12](#_Toc230285535)

# Supplemental Figure 1: Distribution of residuals.


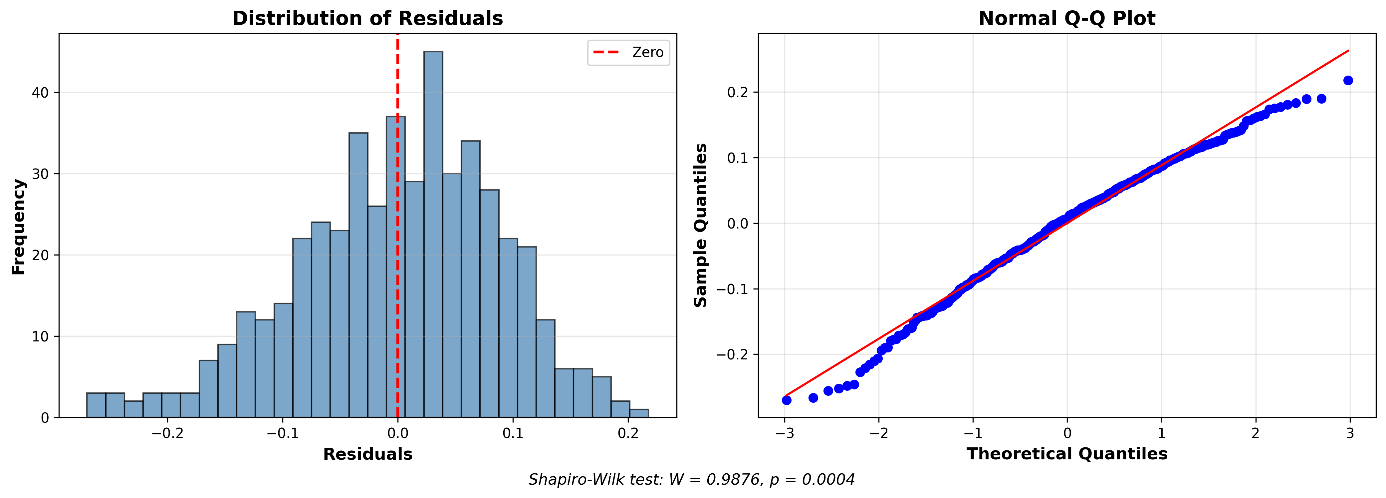


# Supplemental Figure 2: Scatterplot of Residuals vs. Predicted Values


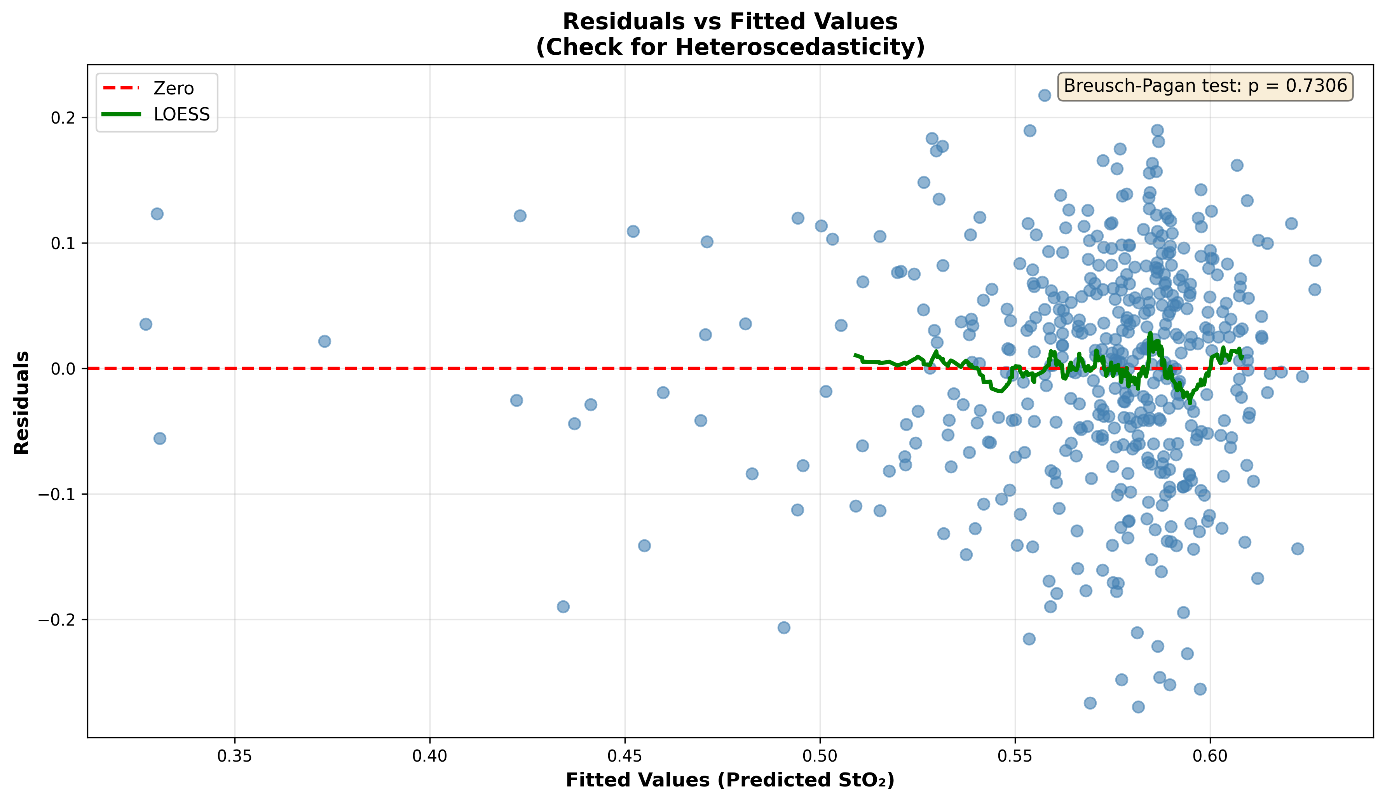


# Supplemental Figure 3: Scatter Plot of Cook's Distance


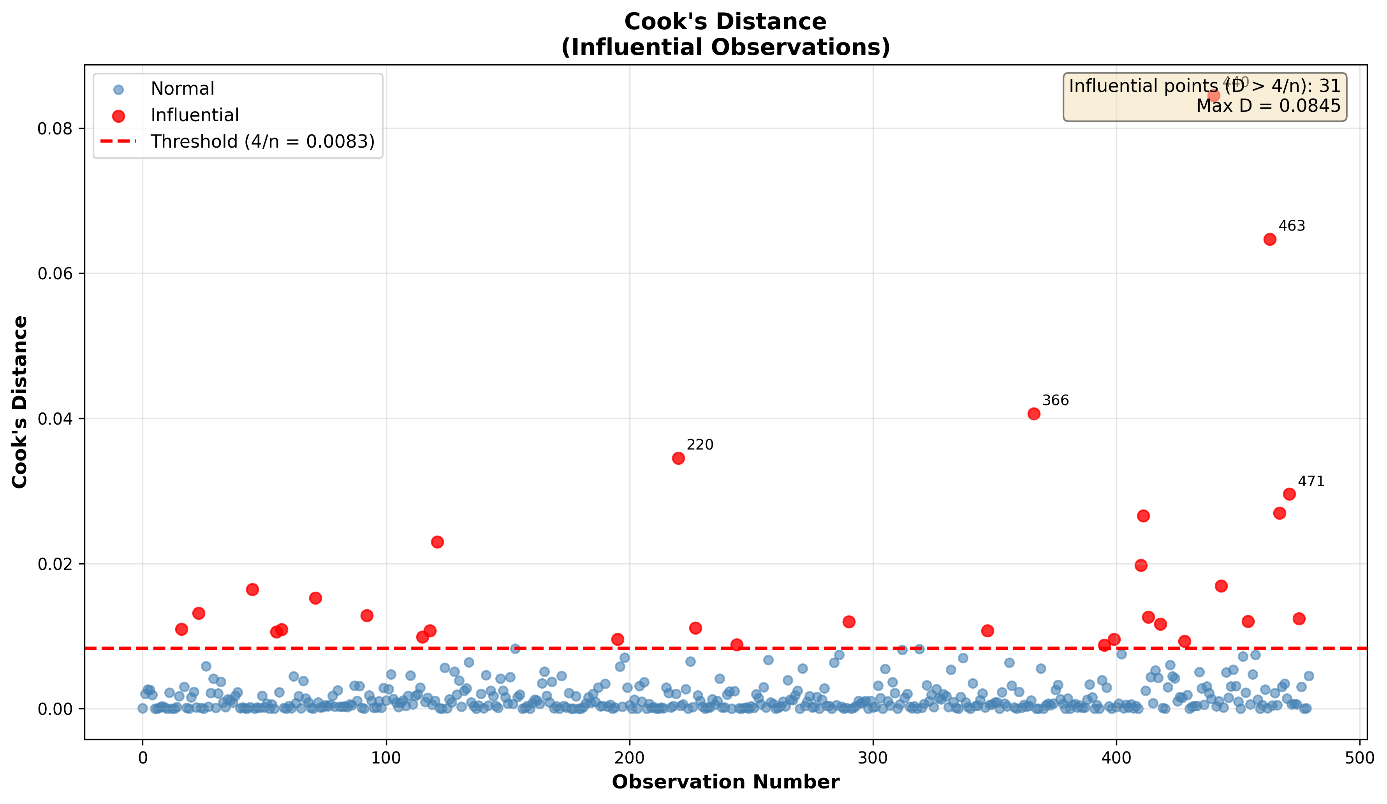


# Supplement Figure 4: 30-Day Mortality across NEE groups


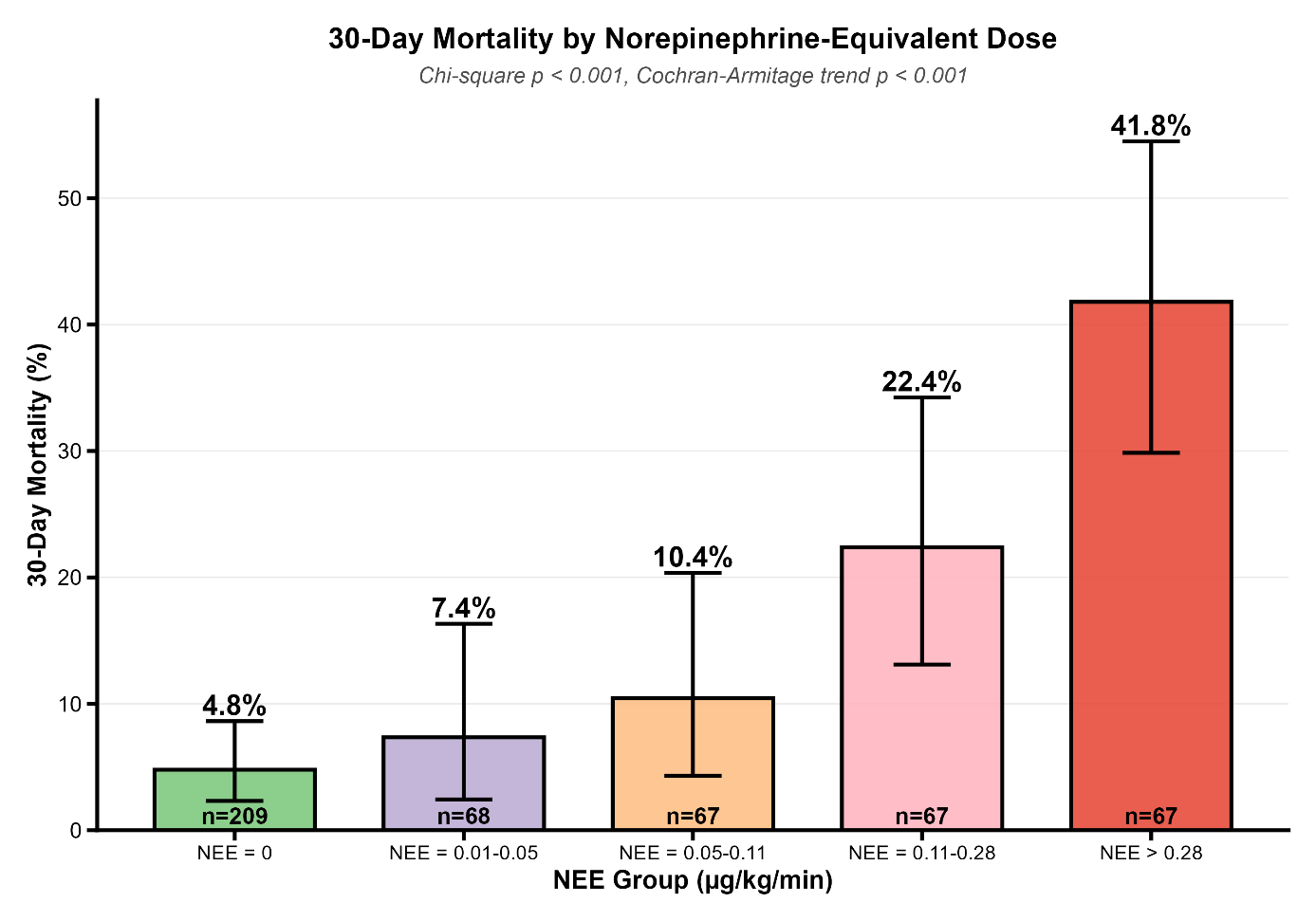


The percentage on top of the error bar represents the mortality rate.

# Supplement Figure 5: StO₂ trajectories from shock onset to reversal stratified by 30-day mortality


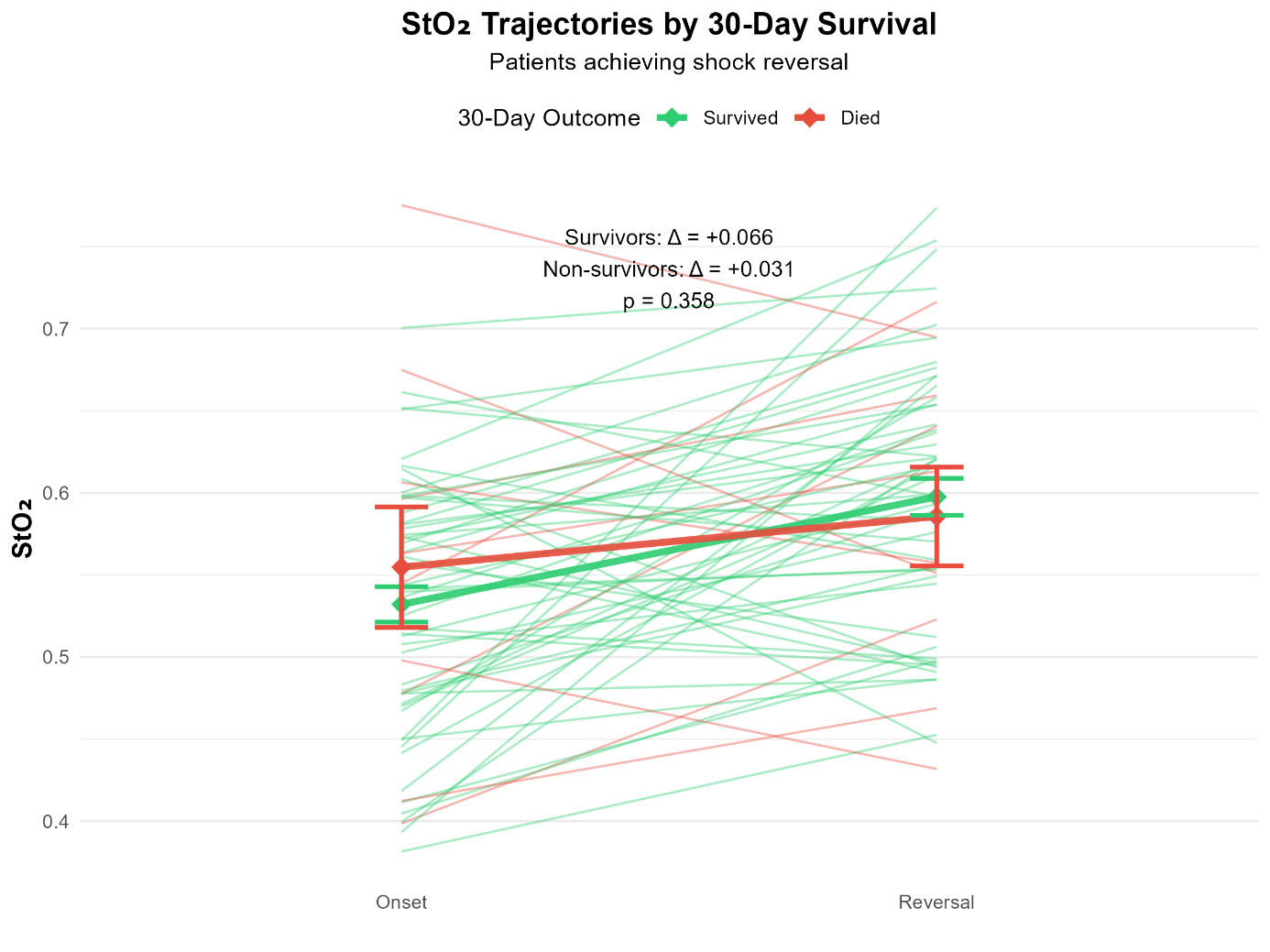


Individual patient trajectories (thin lines) and mean values with standard error (bold lines with error bars) are shown for survivors (green) and non-survivors (red). Survivors demonstrated greater StO₂ improvement (+6.6%) compared to non-survivors (+3.1%), though this difference did not reach statistical significance (p = 0.358, n = 61 patients with complete mortality data).

# Supplemental Table 1: Baseline Characteristics of excluded patients

| Variable | Excluded Patients (n=6) |
| --- | --- |
| Age, years, mean ± SD | 67.7 ± 10.8 |
| Weight, mean ± SD [kg] | N.A. |
| Female sex, n (%) | 0 (0) |
| APACHE II score, mean ± SD | 38.3 ± 5.9 |
| SOFA score, mean ± SD | 9.6 ± 4.0 |
| Preexisting cardiovascular disease, n (%) | 2 (33) |
| CKD, n (%) | 1 (17) |
| Acute kidney injury, n (%) | 2 (33) |
| RRT, n (%) | 2 (33) |
| Malignant disease, n (%) | 1 (17) |
| Ventilated at baseline, n (%) | 6 (100) |
| Fluid Balance Day 1, mean ± SD [ml] | 2389.6 ± 2873.0 |
| Sepsis present, n (%) | 3 (50) |
| Septic shock, n (%) | 1 (17) |
| Unclear Sepsis Status, n (%) | 2 (33) |
| Sepsis focus (among sepsis patients) |  |
| Abdominal, n (%) | 1 (33) |
| Pulmonal, n (%) | 0 (33) |
| UTI, n (%) | 1 (33) |
| Soft tissue/skin, n (%) | 0 (0) |
| Multiple foci, n (%) | 0 (0) |
| Other/unknown, n (%) | 1 (33) |

Values given as mean and standard deviation or absolute and relative frequencies. APACHE: Acute Physiology And Chronic Health Evaluation-Score, SOFA: Sequential Organ Failure Assessment Score, CKD: Chronic Kidney Failure, RRT: Renal Replacement Therapy, UTI: Urinary Tract Infection.

# Supplemental Table 2: Hyperspectral Imaging data, lactate, and creatinine levels according to NEE-quartiles.

| Variable | NEE = 0 (n=221) | NEE 0.01- 0.05 (n=71) | NEE 0.05-0.11 (n=70) | NEE 0.11-0.28 (n=70) | NEE >0.28 (n=70) | p-value (ANOVA) |
| --- | --- | --- | --- | --- | --- | --- |
| StO₂, mean ± SD | 0.59 ± 0.09 | 0.59 ± 0.09 | 0.57 ± 0.08 | 0.55 ± 0.10 | 0.51 ± 0.10 | <0.001 |
| NIR, mean ± SD | 0.48 ± 0.08 | 0.48 ± 0.08 | 0.48 ± 0.07 | 0.47 ± 0.07 | 0.45 ± 0.08 | 0.030 |
| TWI, mean ± SD | 0.67 ± 0.07 | 0.68 ± 0.06 | 0.70 ± 0.07 | 0.72 ± 0.06 | 0.72 ± 0.07 | <0.001 |
| THI, mean ± SD | 0.25 ± 0.10 | 0.24 ± 0.12 | 0.23 ± 0.10 | 0.28 ± 0.13 | 0.29 ± 0.11 | 0.002 |
| Lactate [mmol/L], mean ± SD | 1.51 ± 1.0 | 1.56 ± 1.11 | 1.65 ± 0.98 | 2.56 ± 2.59 | 4.57 ± 5.00 | <0.001 |
| Creatinine [mg/dL], mean ± SD | 1.37 ± 1.42 | 1.27 ± 0.96 | 1.31 ± 1.12 | 1.71 ± 0.96 | 2.01 ± 1.07 | <0.001 |

StO₂: tissue oxygen saturation, NIR: near-infrared index, TWI: tissue water index, THI: tissue hemoglobin index. NEE: Norepinephrine Equivalent (µg/kg/min).

# Supplemental Table 3: Sensitivity analyses assessing non-linearity of the association between NEE and StO₂

| **Model** | **Key parameter** | **Estimate** | **95% CI** | **p-value** | **Model comparison vs linear** | **AIC** |
| --- | --- | --- | --- | --- | --- | --- |
| **Primary linear model** | NEE | -0.093 | -0.150 to -0.036 | 0.001 | Reference | -954.6 |
| **Quadratic model** | NEE² | -0.043 | -0.154 to 0.068 | 0.450 | p = 0.450 | -953.2 |
| **Restricted cubic spline** | Overall NEE effect | — | — | 0.012 | — | — |
| **Restricted cubic spline** | Non-linear component | — | — | 0.693 | — | — |
| **Natural spline model** | Overall spline terms | — | — | — | p = 0.477 | -951.2 |

AIC: Akaike’s information criterion; CI = Confidence interval; NEE = Norepinephrine-equivalent dose

# Supplemental Table 4: Comparison of Standard Errors HC3 and OLS for Linear Regression

| Predictor | OLS | HC3 |
| --- | --- | --- |
| Age (years) | 0.0003 | 0.0003 |
| MAP (mmHg) | 0.0003 | 0.0003 |
| Lactate (mmol/L) | 0.0003 | 0.0002 |
| SOFA | 0.0012 | 0.0012 |
| NEE | 0.0288 | 0.0320 |

OLS: Oridnary Least Squares, HC3: Heteroskedasticity-consistent standard errors

# Supplemental Table 5: Non-Sepsis Subgroup

| Parameter | NEE = 0 (n = 207) | NEE 0.01 –0.03 (n = 43) | NEE 0.03 –0.07 (n = 42) | NEE 0.07 – 0.16 (n = 42) | NEE > 0.16 (n = 42) | p-value (ANOVA) |
| --- | --- | --- | --- | --- | --- | --- |
| StO₂, mean ±SD | 0.588 ± 0.093 | 0.597 ± 0.09 | 0.580 ± 0.09 | 0.556 ± 0.09 | 0.537 ± 0.09 | 0.006 |
| NIR, mean ±SD | 0.474 ± 0.08 | 0.491 ± 0.06 | 0.492 ± 0.06 | 0.484 ± 0.06 | 0.455 ± 0.07 | 0.099 |
| TWI, mean ±SD | 0.670 ± 0.07 | 0.678 ± 0.05 | 0.685 ± 0.07 | 0.690 ± 0.07 | 0.720 ± 0.06 | <0.001 |
| THI, mean ±SD | 0.245 ± 0.10 | 0.222 ± 0.10 | 0.221 ± 0.09 | 0.220 ± 0.10 | 0.272 ± 0.11 | 0.059 |

Significant post-hoc Tukey test for StO₂: NEE=0 vs Q4 (p=0.011), Q1 vs Q4 (p=0.024). Significant post-hoc comparisons for TWI: NEE=0 vs Q4 (p<0.001), Q1 vs Q4 (p=0.035). ). StO₂ = tissue oxygen saturation, NIR = near-infrared index, TWI = tissue water index, THI = tissue hemoglobin index. NEE = norepinephrine-equivalent dose (µg/kg/min).
